# Terbinafine-Induced Liver Injury: A Case Report

**DOI:** 10.7759/cureus.50749

**Published:** 2023-12-18

**Authors:** Misbah Jilani, Syed Zaidi, Marco Cazares Parson, Michael J Brockman, Nawar Hakim, Brian P Edwards

**Affiliations:** 1 Internal Medicine, Texas Tech University Health Sciences Center El Paso, El Paso, USA; 2 Pathology, Texas Tech University Health Sciences Center El Paso, El Paso, USA

**Keywords:** drug-induced liver injury, cholestatic liver injury, liver toxicity, conjugated hyperbilirubinemia, terbinafine

## Abstract

Hepatic injuries attributable to terbinafine usage are a well-documented yet infrequent phenomenon. This case study details the clinical presentation and management of a 70-year-old Hispanic female, with no previous medical history, subsequently hospitalized for progressive jaundice, right upper quadrant abdominal discomfort, and worsening pruritus. A comprehensive review of her prior records revealed a recent terbinafine prescription for onychomycosis, which she took consistently for five weeks and then self-discontinued four weeks before her current admission. Laboratory tests on admission revealed a cholestatic pattern of liver injury, evident by transaminitis and conjugated hyperbilirubinemia. The R factor used to determine whether a liver injury is hepatocellular or cholestatic was 0.9. Further diagnostic imaging, including abdominal ultrasound, CT of the abdomen, and magnetic resonance cholangiopancreatography, failed to disclose an obstructive pathology, revealing only cholelithiasis and chronic cholecystitis. Therapeutically, the patient was initiated on hydroxyzine to address symptoms of pruritus, and then subsequently underwent a liver biopsy. Histopathologic findings from the biopsy revealed benign hepatic parenchyma demonstrating focal canalicular cholestasis, mild chronic inflammation involving select portal tracts, and chronic lobular inflammation, suggesting terbinafine-induced hepatotoxicity. This case highlights the challenges of diagnosing terbinafine-induced liver injury, emphasizing the need for a high index of clinical suspicion and recognizing the potential for prolonged symptomatic manifestation after drug discontinuation. This article provides valuable insights into the complexities inherent in such diagnoses and significantly enriches a medical provider’s approach to diagnosing and treating unexplained liver injuries.

## Introduction

Terbinafine is a frequently prescribed antifungal medication primarily used for the treatment of onychomycosis. Its mechanism of action involves non-competitive inhibition of the enzyme squalene epoxidase, leading to fungal cell death [1]. Commonly reported adverse effects of terbinafine include gastrointestinal disturbances, dysgeusia, rash, and headache [2]. Hepatotoxicity is an infrequent side effect with an incidence of 1 in 50,000 to 120,000 [3]. The pathophysiology behind this injury is not well researched, with only a few case reports published, making this condition notably challenging to diagnose and manage. In this case report, we detail the case of a 70-year-old Hispanic female who presented with progressive jaundice, pruritus, and right upper quadrant abdominal pain. Despite presenting symptoms and lab findings suggestive of obstructive jaundice, imaging failed to disclose an obstructive pathology; however, the liver biopsy was compatible with manifestations of hepatotoxicity secondary to terbinafine exposure, suggesting a drug-induced liver injury from the patient’s recent use of terbinafine.

The diagnosis of terbinafine-induced liver injury can be challenging owing to its variable clinical manifestations and the potential for prolonged symptomatic presentations despite discontinuation. While the majority of documented cases indicate recovery within 2-12 months post-discontinuation, there are isolated incidents where the injury progressed to liver failure requiring transplantation [2]. This case report emphasizes the importance for clinicians to identify potential life-threatening causes of liver disease while maintaining a high index of suspicion for drug-induced injury, as a delay in diagnosis can result in severe consequences.

## Case presentation

A 70-year-old Hispanic female, with no significant past medical history, was admitted to the hospital for further evaluation of one month of progressive jaundice. She initially presented with complaints of worsening right upper quadrant pain, pruritus, and generalized jaundice for one month. She also reported concurrent dysgeusia, decreased appetite, acholic stools, dark urine, nausea, and vomiting. Notably, her recent history included the use of oral terbinafine for onychomycosis prescribed by her primary care physician. She adhered to a five-week regimen and then discontinued the medication after developing jaundice and pruritus one month before the current hospitalization. She denied any other medication, herbal supplement, alcohol use, or illicit drug use. At the time of admission, her vital signs were stable. She was afebrile and normotensive with a blood pressure of 130/76 mmHg, a heart rate of 62 beats per minute, and a respiratory rate of 17 breaths per minute. Physical examination was notable for pronounced jaundice, scleral icterus, and generalized tenderness to palpation of the abdomen.

Initial laboratory workup was significant for elevated aspartate aminotransferase (AST), alanine aminotransferase (ALT), alkaline phosphatase (ALP), total bilirubin, and direct bilirubin, with an R factor of 0.9, consistent with a cholestatic pattern of liver injury (Table 1). Viral workup investigating hepatitis, Epstein-Barr virus, and cytomegalovirus was unremarkable. The toxicology screen was negative. Autoimmune workup including serum IgG, IgM, antinuclear antibody, and antimitochondrial antibody were negative, while a low amount (26, normal range <20) of anti-smooth muscle antibody was detected.

**Table 1:** Significant laboratory values on admission. AST: aspartate aminotransferase; ALT: alanine aminotransferase; WBC: white blood count

Tests	Results	Normal Range
AST	69 IU/L	17–59 IU/L
ALT	64 IU/L	0–50 IU/L
Alkaline phosphatase	238 IU/L	38–126 IU/L
Total bilirubin	24.5 mg/dL	0.2–1.3 mg/dL
Direct bilirubin	21.50 mg/dL	0.0–0.3 mg/dL
WBC	4.76 U/L	4,500–1,000 U/L
Hemoglobin	12.7 g/dL	12.0–15.0 g/dL
Hematocrit	37.4%	36.0–47.0%

**Figure 1:**
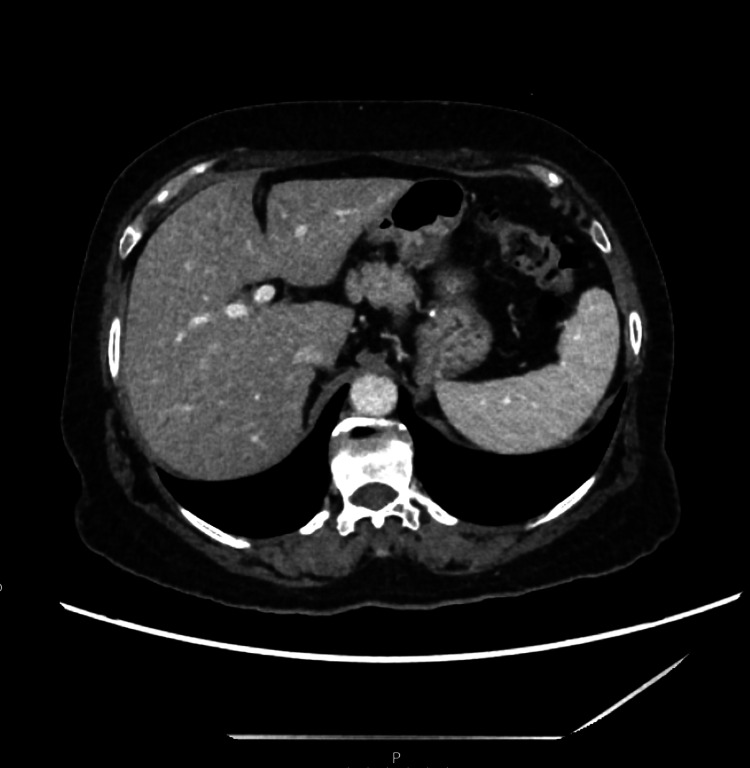
Computed tomography of the abdomen with contrast revealing no acute findings.

**Figure 2:**
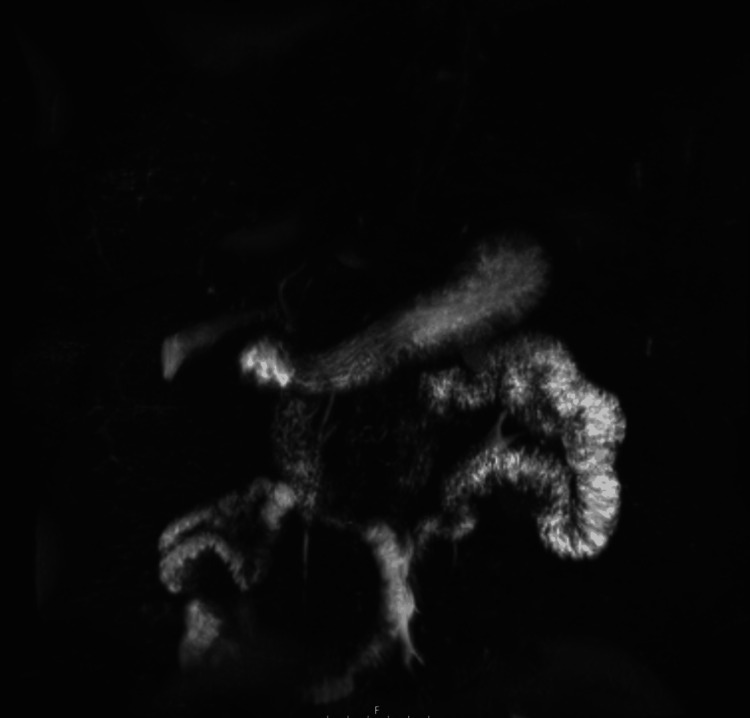
Magnetic resonance cholangiopancreatography without any evidence of choledocholithiasis or biliary obstruction.

Abdominal ultrasound showed cholelithiasis and gallbladder sludge, and CT abdomen (Figure 1) and MRI abdomen revealed similar findings. Magnetic resonance cholangiopancreatography to further evaluate demonstrated findings suggestive of chronic cholecystitis without any evidence of choledocholithiasis or biliary obstruction (Figure 2).

**Figure 3:**
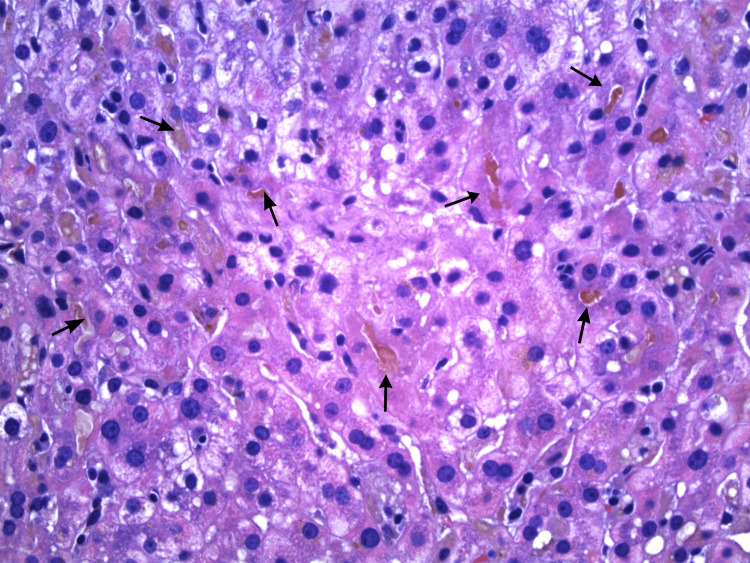
Hepatic parenchyma demonstrates multiple foci of canalicular cholestasis with cholestatic bile plugs (arrows). Hematoxylin and eosin stain, original magnification ×400.

**Figure 4:**
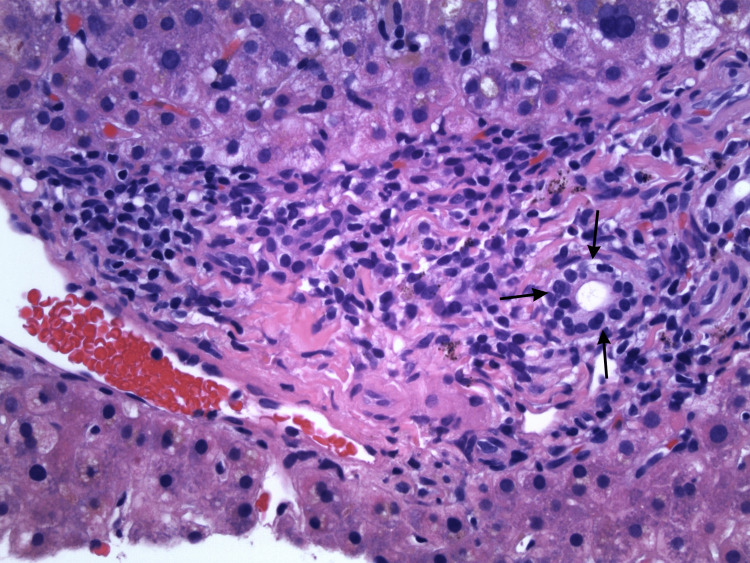
Portal tract expanded by chronic inflammation. The arrows point to the bile ductule. Hematoxylin and eosin stain, original magnification ×400.

**Figure 5:**
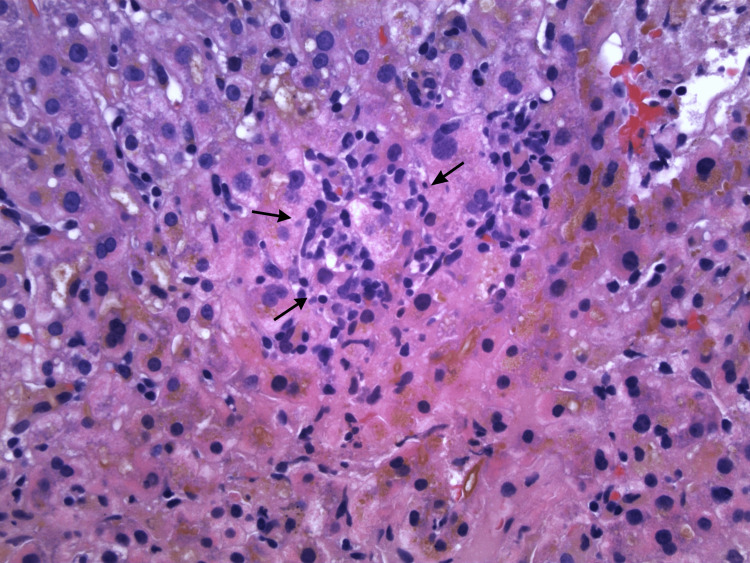
Lobular inflammation with degenerated hepatocytes (arrows). Hematoxylin and eosin stain, original magnification ×400.

Imaging-guided core needle biopsy of the liver was performed and revealed benign hepatic parenchyma demonstrating focal canalicular cholestasis (Figure 3), focal mild chronic inflammation involving some portal tracts (Figure 4), and focal lobular chronic inflammation (Figure 5). Trichrome stain was negative for bridging fibrosis or cirrhosis; periodic acid-Schiff with diastase stain did not show intracytoplasmic inclusions; reticulin stain demonstrated intact hepatic framework; and Prussian blue stain showed no increase in hepatic iron. The non-specific pattern histologically presented in the liver biopsy in combination with the clinical picture led to a diagnosis of terbinafine-induced liver injury.

The pruritus was managed using hydroxyzine, and upon discharge, the patient noted an alleviation of the initial symptoms. The patient was instructed to not resume terbinafine and follow up outpatient with gastroenterology in two weeks. At the six-week follow-up, the patient’s lab results demonstrated significant improvement, with a total bilirubin level of 4.5 mg/dL and a direct bilirubin level of 3.60 mg/dL.

## Discussion

This patient’s hospitalization emphasizes the challenges involved in diagnosing terbinafine-induced liver injury as its presentation mirrors that of other hepatobiliary diseases. As demonstrated in this case report, it frequently manifests with non-specific symptoms of jaundice, abdominal pain, nausea, and vomiting [4]. This aligns with previous research highlighting the diagnostic difficulty of drug-induced liver injury, where clinical presentation often lacks specificity [5]. Drug-induced liver injury usually exhibits a cholestatic pattern of injury, as denoted by the R factor, where an R-value of less than 2 indicates cholestatic injury and an R-value of greater than 5 suggests hepatocellular injury [6].

In 2017, a critically appraised topic on the clinical presentation of terbinafine-induced liver injury examined 173 cases. The analysis revealed that 31 of these cases reported jaundice. The average age of affected patients was 54 years, ranging from 24 to 75 years, and the onset of symptoms occurred within an average of 33 days, ranging from 5 to 84 days [3]. Most patients develop symptoms within six months of exposure to the offending agent [1]. As demonstrated by this case, discontinuing medication does not immediately lead to symptom relief. Current data suggests complete resolution usually takes three to six months after medication discontinuation, though some cases might extend to 12 months [1]. Notably, severe outcomes including liver failure are possible, especially in those with continued use after the development of symptoms [6]. Thus, prompt recognition and discontinuation of the causative agent are crucial for achieving favorable outcomes.

Furthermore, it is imperative to obtain a thorough patient history and maintain a high clinical index of suspicion when treating patients with non-specific biliary findings. Our patient did not initially reveal the use of terbinafine, prompting extensive diagnostic testing. It was only through additional investigation into her medical history that we discovered her prior use of terbinafine. The identification of the use of a potentially hepatotoxic drug, however, does not immediately lead to diagnosis, as drug-induced liver injury is a diagnosis of exclusion. Other causes of cholestasis, such as choledocholithiasis, benign bile duct stricture, pancreatic cancer, cholangiocarcinoma, and autoimmune conditions should also be excluded before a definitive diagnosis is made. As illustrated by this case, a comprehensive patient history is pivotal in excluding alternative causes of cholestasis and preventing treatment delays. Clinicians must obtain a detailed patient history, including the use of any medications, herbal products, or supplements. While terbinafine remains an effective treatment for onychomycosis, clinicians should be vigilant in monitoring patients and promptly addressing any clinical signs of hepatic involvement to ensure patient safety and optimal therapeutic outcomes.

## Conclusions

Terbinafine-induced liver injury presents as a multifaceted condition demanding a comprehensive evaluation encompassing the patient’s clinical, laboratory, imaging, and biopsy findings. The absence of distinct symptoms and specific lab markers adds to the complexity of diagnosis. Swift cessation of the implicated agent remains critical in attaining symptomatic resolution and averting further hepatic damage. This case report highlights the significance of thoroughly gathering patient history, as well as ruling out alternative etiologies of cholestasis before arriving at a conclusive diagnosis. It also emphasizes the necessity of monitoring for clinical signs of liver dysfunction in patients taking terbinafine, with rapid discontinuation when needed.
